# 1142. Impact of Informational Vaccine Notices Sent to Parents Prior to their Child’s 11^th^ Birthday on Receipt of Adolescent Vaccines

**DOI:** 10.1093/ofid/ofad500.983

**Published:** 2023-11-27

**Authors:** Kayla E Hanson, Meranda Eggebrecht, Penny Funk, Ben Christianson, Edward Belongia, Jeffrey J VanWormer, Charnetta L Williams, Huong McLean

**Affiliations:** Marshfield Clinic Research Institute, Marshfield, Wisconsin; Marshfield Clinic Health System, Marshfield, Wisconsin; Marshfield Clinic Health System, Marshfield, Wisconsin; Minnesota Department of Health, St. Paul, Minnesota; Marshfield Clinic Research Institute, Marshfield, Wisconsin; Marshfield Clinic Research Institute, Marshfield, Wisconsin; Centers for Disease Control and Prevention, Atlanta, Georgia; Marshfield Clinic Research Institute, Marshfield, Wisconsin

## Abstract

**Background:**

Several vaccines, including human papillomavirus (HPV), tetanus-diphtheria-acellular pertussis vaccine (Tdap), and meningococcal serogroup ACWY vaccine (MenACWY), are recommended at age 11-12 years. In the United States, uptake of these vaccines, particularly HPV, is suboptimal. Systems to identify and notify patients who are coming due or overdue for vaccinations (reminder/recall) have been shown to be an effective strategy to increase vaccination. We assessed the impact of sending informational vaccine notices to parents of 10-year-old patients on receipt of adolescent vaccines in a predominantly rural, regional healthcare system in northcentral Wisconsin.

**Methods:**

10-year-old patients with an upcoming 11^th^ birthday (i.e., aged 10 years, 10 months or 10 years, 11 months) were identified at Marshfield Clinic Health System on a monthly basis and were randomized 1:1 to intervention (informational vaccine notice sent to the patient’s parent) vs. usual care (no notice). Randomization was stratified by rurality (rural, nonrural; defined by Rural-Urban Commuting Area Codes) and the parent’s preferred communication method (letter, text message, email). Receipt of adolescent vaccines (HPV, Tdap, MenACWY), as well as receipt of seasonal influenza vaccine and COVID-19 vaccine primary series, was assessed during the 90 days after randomization.

**Results:**

From August 2021 through May 2022, 2,266 10-year-old patients were randomized (1,122 to the intervention and 1,144 to usual care) and 1,122 notices were sent to parents (576 letters, 544 text messages, and 2 emails). The majority of patients were non-Hispanic white (82%), lived in a rural area (63%), and approximately half were female (51%). Demographics did not vary by study arm. Receipt of HPV, Tdap, and MenACWY was 3.7 to 4.4 percentage points higher among patients randomized to the intervention vs. usual care (Table 1). No difference in uptake was observed for COVID-19 or influenza vaccines.

**Figure:**
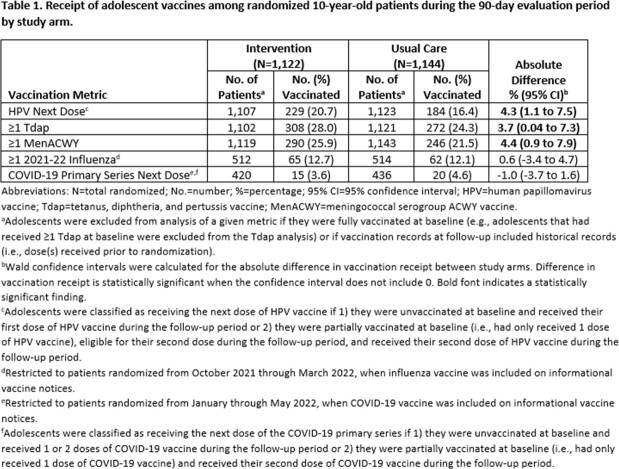

**Conclusion:**

Informational notices sent to parents prior to their child’s 11^th^ birthday resulted in modestly higher uptake of adolescent vaccines. Healthcare systems should consider sending vaccination notices, mailed or electronic, to parents whose children are coming due for recommended vaccines.

**Disclosures:**

**Kayla E. Hanson, MPH**, Seqirus: Grant/Research Support **Edward Belongia, MD**, Seqirus: Grant/Research Support **Huong McLean, PhD, MPH**, Seqirus: Grant/Research Support

